# Effect of Postoperative Atrial Fibrillation After Cardiac Surgery: A Meta‐Analysis

**DOI:** 10.1002/clc.70053

**Published:** 2024-12-04

**Authors:** Fangzhou Qu, Wei Yang, Ni He, Shangcheng Qu, Xiao Zhou, Huayan Ma, Xin Jiang

**Affiliations:** ^1^ Medical School Xizang Minzu University Xianyang China; ^2^ Emergency Department The Affiliated Hospital of Xizang University for Nationalities Xianyang China; ^3^ Department of Cardiology Shaanxi Provincial People's Hospital Shaanxi China; ^4^ School of Mechanic Engineering Sichuan University Sichuan China; ^5^ Department of Dermatology The Second Xiangya Hospital of Central South University Changsha China

**Keywords:** cardiac surgery, mortality at 1 year, mortality at 10 year, mortality at 5 year, overall stroke, postoperative atrial fibrillation

## Abstract

**Background:**

A meta‐analysis study was conducted to determine how to predict the effect of postoperative atrial fibrillation after cardiac surgery.

**Hypothesis:**

Long‐term mortality and cardiovascular morbidity are linked to postoperative atrial fibrillation.

**Method:**

Until August 2024, a comprehensive literature study was completed, and 3486 connected studies were revised. The 38 selected studies included 241 299 cardiac surgery participants at the beginning of the study. The odds ratio (OR) and 95% confidence intervals (CIs) were used to look at the effect of atrial fibrillation after heart surgery using two‐sided methods and either a fixed or random model.

**Results:**

Individuals with cardiac surgery with postoperative atrial fibrillation had significantly higher mortality at 1 year (OR, 1.39; 95% CI, 1.12–1.72, *p* < 0.001), mortality at 5 years (OR, 1.61; 95% CI, 1.33–1.94, *p* < 0.001), mortality at 10 years (OR, 1.61; 95% CI, 1.39–1.87, *p* < 0.001), and overall stroke (OR, 1.61; 95% CI, 1.34–1.94, *p* < 0.001) compared to without postoperative atrial fibrillation.

**Conclusions:**

Individuals with cardiac surgery with postoperative atrial fibrillation had significantly higher mortality at 1 year, mortality at 5 years, mortality at 10 years, and overall stroke compared to those without postoperative atrial fibrillation. To validate this discovery, more research and caution must be implemented when interacting with its values.

## Summary

- Postoperative atrial fibrillation is the most frequent side effect following heart surgery. Although postoperative atrial fibrillation occurs in 20%–40% of individuals following heart surgery, its effect on long‐term results following heart surgery is not fully established.
- Individuals with cardiac surgery with postoperative atrial fibrillation had significantly higher mortality at 1 year, mortality at 5 years, mortality at 10 years, and overall stroke compared to those without postoperative atrial fibrillation.
- To validate this discovery, more research and caution must be implemented when interacting with its values.

## Graphical abstract

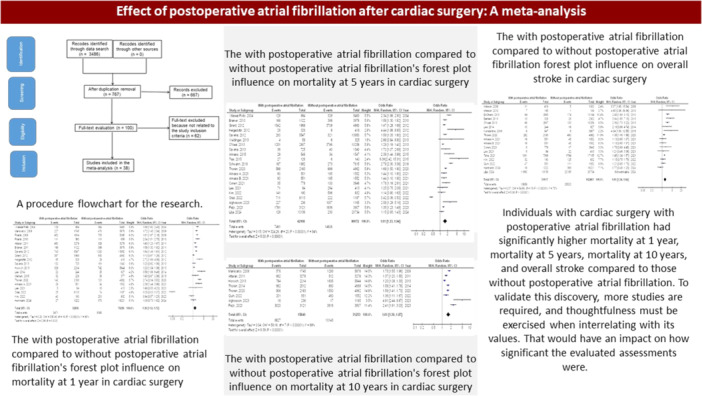

## 1 Backgrounds

Postoperative atrial fibrillation is the most frequent side effect following heart surgery. Although postoperative atrial fibrillation occurs in 20%–40% of individuals following heart surgery, its effect on long‐term results following heart surgery is not fully established [1]. Short‐term consequences of postoperative atrial fibrillation include longer hospital stays, a higher frequency of thromboembolic events, and a higher rate of in‐hospital mortality [2]. Before being released from the hospital, the majority of patients spontaneously return to sinus rhythm. Rate and rhythm control are the mainstays of postoperative atrial fibrillation therapy, with anticoagulation being used sparingly [3]. New evidence has challenged historical notions that post‐cardiac surgery postoperative atrial fibrillation is benign and self‐limiting. It shows that individuals with postoperative atrial fibrillation have a fivefold greater chance of developing persistent atrial fibrillation [3]. There is a correlation between postoperative atrial fibrillation and death and ischemic stroke that lasts for up to 10 years following surgical intervention, according to two recent meta‐analyses looking at the long‐term effects of postoperative atrial fibrillation following coronary artery bypass grafting [4]. There has not been a recent comprehensive analysis of the literature regarding postoperative atrial fibrillation's long‐term effects on patients who have heart surgery in general. Following heart surgery, we carried out a meta‐analysis of the long‐term mortality and cardiovascular morbidity linked to postoperative atrial fibrillation.

### 1.1 Objectives

We used a meta‐analysis to assess the effect of postoperative atrial fibrillation after cardiac surgery.

### 1.2 Methods

#### 1.2.1 Eligibility Criteria

To provide an overview, of the studies that showed the effect of postoperative atrial fibrillation after cardiac surgery [5].

#### 1.2.2 Information Sources

**Figure 1:**
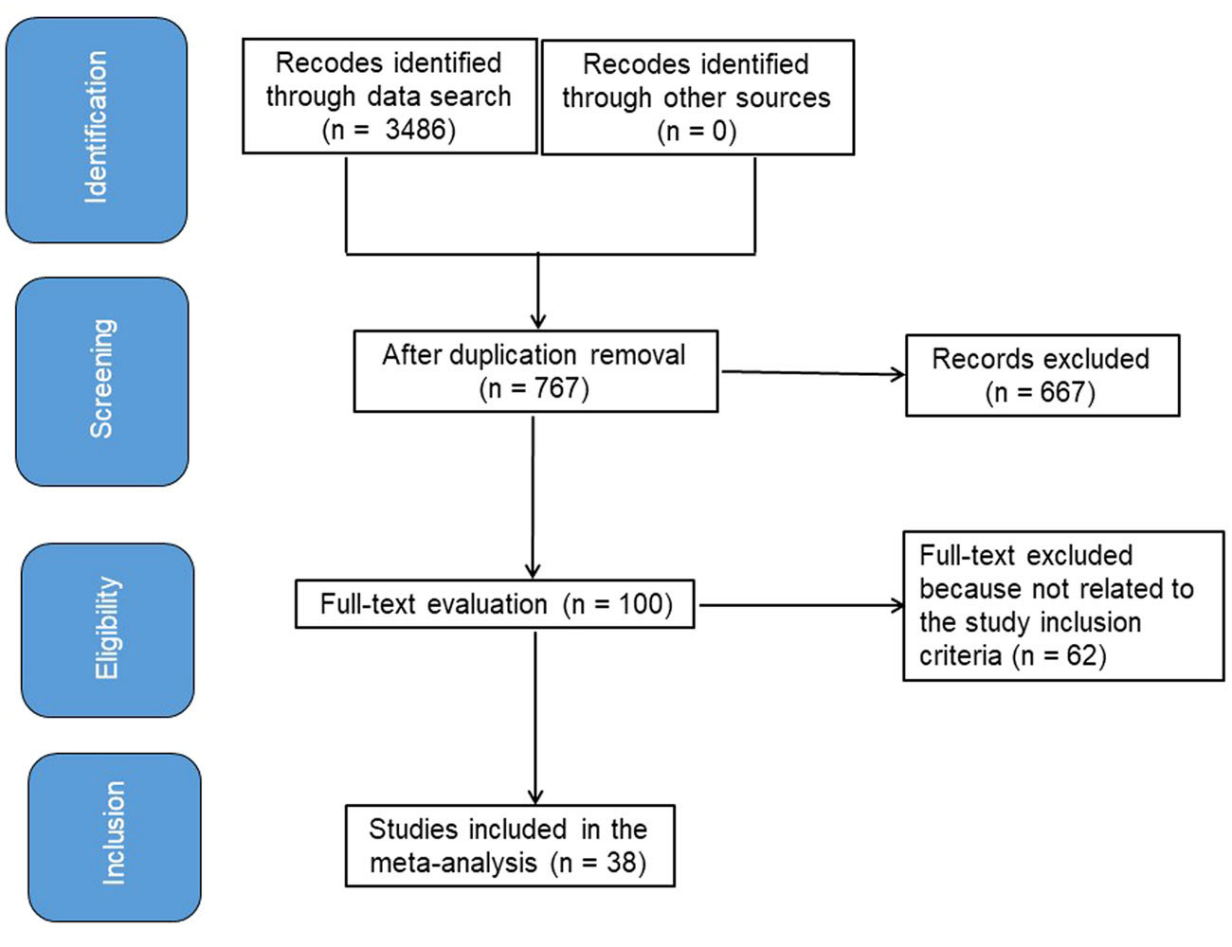
A procedure flowchart for the research.

Figure 1 represents the entirety of the study. When the following inclusion criteria were satisfied, the literature was incorporated into the study [6, 7]:

1. The study was a randomized controlled trial (RCT), observational, prospective, or retrospective study.
2. The people who were chosen for investigation had cardiac surgery.
3. Postoperative atrial fibrillation was integrated into the intervention.
4. The study made a distinction about the effect of postoperative atrial fibrillation after cardiac surgery.

Studies that did not check the effect of postoperative atrial fibrillation after cardiac surgery, studies on individuals without postoperative atrial fibrillation, and studies with no comparison significance were also omitted [8, 9].

### 1.3 Search Strategy

A search protocol process was identified using the PICOS view, and we defined it as follows: the “population” consisted of people with cardiac surgery, P; Postoperative atrial fibrillation was the “intervention” or “exposure,” and the “comparison” involved correlation between with postoperative atrial fibrillation and without postoperative atrial fibrillation variables; the “outcome” was the effect on mortality, overall stroke; and the “research design” was without boundaries [10].

We have thoroughly searched the databases of Google Scholar, Embase, the Cochrane Library, PubMed, and OVID through August 2024 using a set of keywords and additional terms as shown in Supporting Information S2: Table 1 [11, 12]. To prevent the inclusion of a study that was unable to establish a link between the effect of postoperative atrial fibrillation after cardiac surgery, the replications of the papers were eliminated, the rest were assembled into an EndNote file, and their titles and abstracts were once again assessed [13, 14].

### 1.4 Selection Process

The meta‐analysis method was then used to organize and assess the process that followed the epidemiological proclamation [15, 16].

### 1.5 Data Collection Process

Some of the criteria utilized to gather data were the name of the first author, research data, research year, nation or region, population type, categories, quantitative and qualitative estimation methods, data sources, outcome estimation, medical and therapy physiognomies, and statistical analysis [17].

### 1.6 Data Items

When a study yielded differing values, we independently gathered the data found on a valuation of the effect of postoperative atrial fibrillation after cardiac surgery.

### 1.7 Research Risk of Bias Assessment

Two authors investigated the potential for bias in the studies and the standard of approaches utilized in papers elected for supplementary analysis. They conducted unbiased reviews of the techniques used for each test.

### 1.8 Effect Measures

Sensitivity analysis was limited to studies that assessed and documented the effect of postoperative atrial fibrillation after cardiac surgery. A subclass analysis was used to compare the correlation between with and without postoperative atrial fibrillation in different patients' variables in cardiac surgery individuals' sensitivity.

### 1.9 Synthesis Methods

Using a dichotomous approach and a random or fixed‐effect model, the odds ratio (OR) and a 95% confidence interval (CI) were determined. A range of 0% to 100% was used to determine the *I* ^2^ index. At 0%, 25%, 50%, and 75% of the data, respectively, there was no, low, moderate, and significant heterogeneity visible [18]. To ensure that the exact model was used, additional structures that show a high degree of similarity with the related inquiry were also examined. The fixed‐effect was selected if *I* ^2^ was less than 50%; otherwise, the random effect was used [18]. A subclass analysis was performed by splitting the original estimation into the previously specified consequence groups. A *p*‐value of less than 0.05 was utilized in the analysis to define the statistical significance of differences across subcategories.

### 1.10 Reporting Bias Assessment

Both quantitative and qualitative methods were employed to measure the bias in the investigations: The Egger regression test and funnel plots, which display the logarithm of the ORs against their standard errors. The presence of investigation bias was determined by *p* ≥ 0.05 [19].

### 1.11 Certainty Assessment

We looked at each p‐value with two‐tailed testing. Graphs and statistical analyses were created using Reviewer Manager Version 5.3 (The Nordic Cochrane Centre, the Cochrane Collaboration, Copenhagen, Denmark).

## 2 Results

Out of 3486 connected studies, 38 papers that were published between 2018 and 2024 and satisfied the inclusion criteria were selected for the study [20, 21, 22, 23, 24, 25, 26, 27, 28, 29, 30, 31, 32, 33, 34, 35, 36, 37, 38, 39, 40, 41, 42, 43, 44, 45, 46, 47, 48, 49, 50, 51, 52, 53, 54, 55, 56]. Supporting Information S2: Table 2 provides access to the findings of these inquiries. At the beginning of the studies that were used, there were 241 299 cardiac surgery participants. There were between 271 and 30 870 subjects as a sample size.

**Figure 2:**
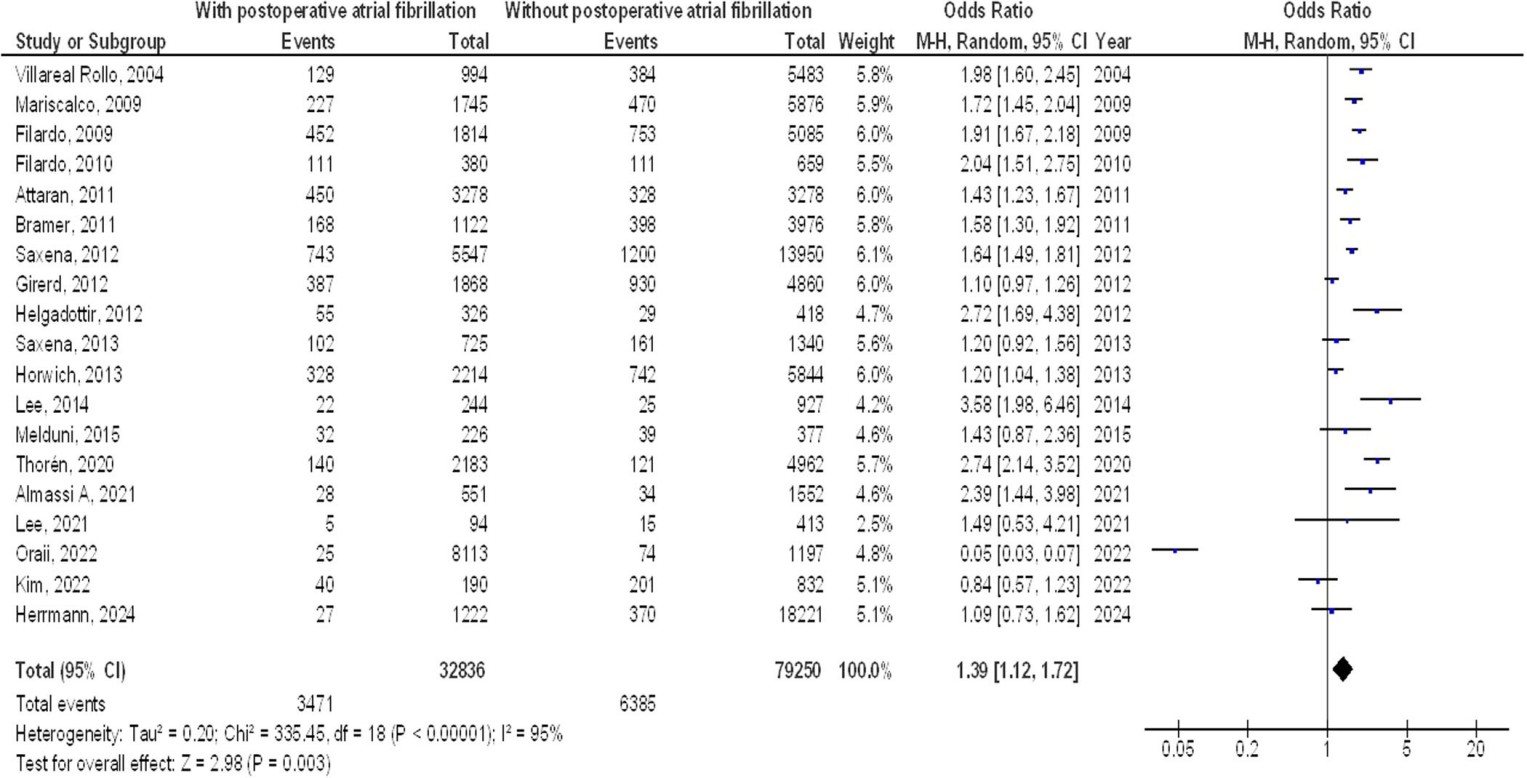
Forest plot of comparison of influence on mortality after 1 year of cardiac surgery of postoperative atrial fibrillation and no postoperative atrial fibrillation.

**Figure 3:**
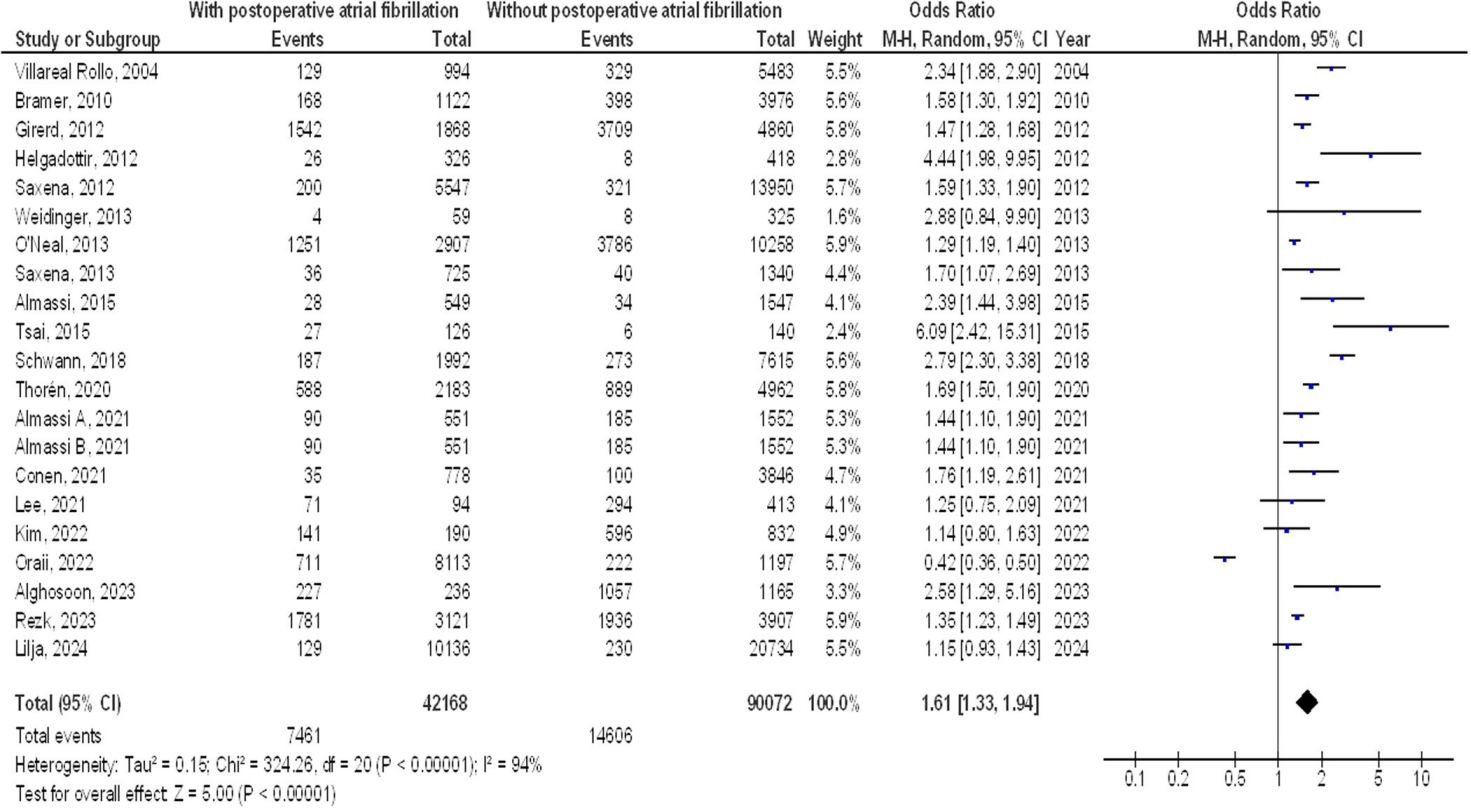
Forest plot of comparison of influence on mortality after 5 years of cardiac surgery of postoperative atrial fibrillation and no postoperative atrial fibrillation.

**Figure 4:**
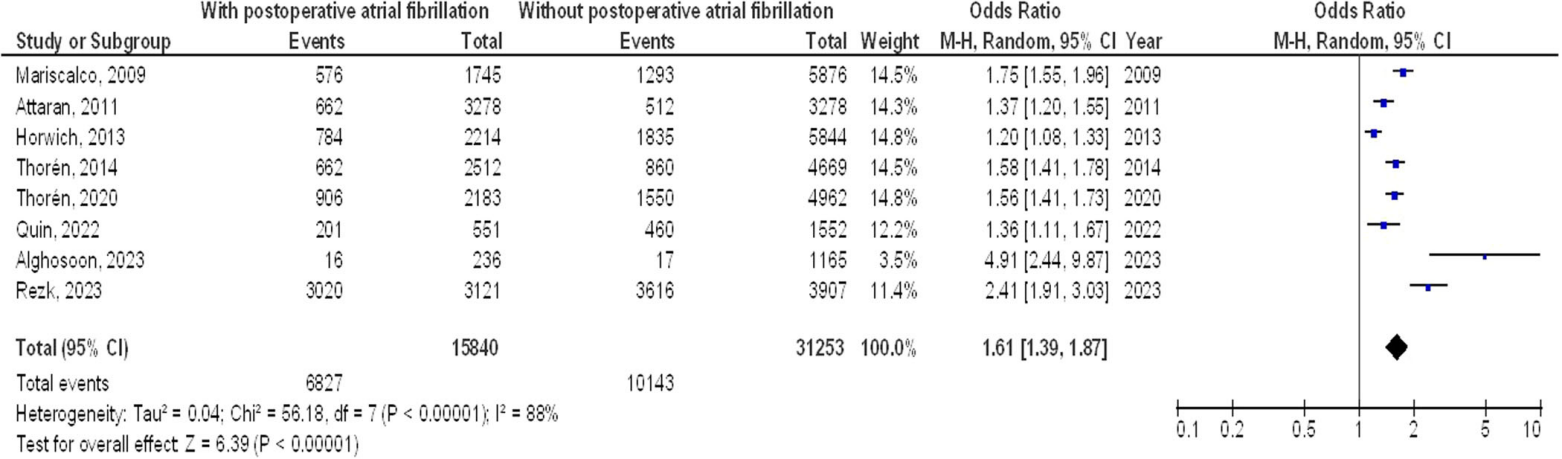
Forest plot of comparison of influence on mortality after 10 years of cardiac surgery of postoperative atrial fibrillation and no postoperative atrial fibrillation.

**Figure 5:**
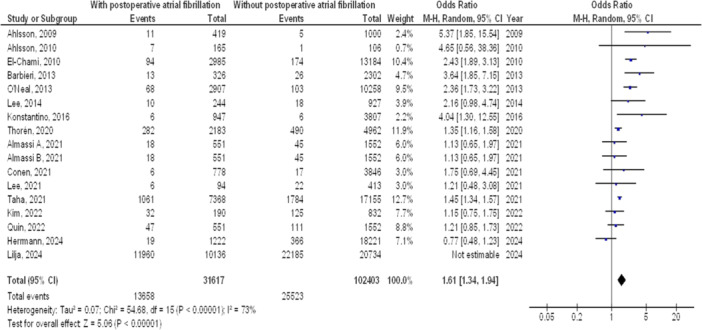
The with postoperative atrial fibrillation compared to without postoperative atrial fibrillation forest plot influence on overall stroke in cardiac surgery.

As illustrated in Figures 2, 3, 4, 5, Individuals with cardiac surgery with postoperative atrial fibrillation had significantly higher mortality at 1 year (OR, 1.39; 95% CI, 1.12–1.72, *p* < 0.001) with high heterogeneity (*I* ^2^ = 95%), mortality at 5 years (OR, 1.61; 95% CI, 1.33–1.94, *p* < 0.001) with high heterogeneity (*I* ^2^ = 94%), mortality at 10 years (OR, 1.61; 95% CI, 1.39–1.87, *p* < 0.001) with high heterogeneity (*I* ^2^ = 88%), and overall stroke (OR, 1.61; 95% CI, 1.34–1.94, *p* < 0.001) with moderate heterogeneity (*I* ^2^ = 73%) compared to without postoperative atrial fibrillation.

The insufficiency of data, for example, age, ethnicity, and gender, on comparative results precluded the application of stratified models to investigate the impacts of particular components. Using the quantitative Egger regression test and the visual interpretation of the funnel plot, no evidence of research bias was detected (*p* = 0.89). However, it was shown that there was no bias in the selective reporting and that the majority of concerned RCTs had poor technical quality.

## 3 Discussions

About 241 299 cardiac surgery participants were at the starting point of the studies that were utilized for the meta‐analysis [20, 21, 22, 23, 24, 25, 26, 27, 28, 29, 30, 31, 32, 33, 34, 35, 36, 37, 38, 39, 40, 41, 42, 43, 44, 45, 46, 47, 48, 49, 50, 51, 52, 53, 54, 55, 56]. Individuals with cardiac surgery with postoperative atrial fibrillation had significantly higher mortality at 1 year, mortality at 5 years, mortality at 10 years, and overall stroke compared to those without postoperative atrial fibrillation. To validate this discovery, more studies are required, and thoughtfulness must be exercised when interrelating with its values. That would have an impact on how significant the evaluated assessments were [57, 58, 59, 60, 61, 62, 63, 64, 65, 66, 67].

Why patients who undergo cardiac surgeries and develop new‐onset postoperative atrial fibrillation afterward have worse results is unknown. One theory is that postoperative atrial fibrillation is a proxy for higher mortality since it occurs in sicker, frailer patients. Additional evaluation of this theory would be possible with a more thorough grasp of the pathophysiology and risk factors for postoperative atrial fibrillation. Nonetheless, epidemiologic research is the source of the present risk prediction models for postoperative atrial fibrillation; pathophysiologic mechanisms are not the foundation of these models. These models are rarely employed in clinical settings due to their, at best, modest accuracy [68]. We might be able to make more accurate risk prediction models for atrial fibrillation after surgery, as well as for illness and death after heart surgery, if we understand more about how atrial fibrillation happens after surgery. Such models could allow the targeted use of current preventative therapies, such as corticosteroids and antiarrhythmics, in patients most at risk of developing postoperative atrial fibrillation. On the other hand, it is plausible that persistent or recurrent atrial fibrillation following surgery leads to a higher risk of death from cardioembolic stroke. Even though sinus rhythm is restored before hospital discharge, a new meta‐analysis indicates that up to 25% of patients may experience a recurrence of postoperative atrial fibrillation within 4 to 6 weeks [2]. Recurrence of postoperative atrial fibrillation may be able to explain the association between postoperative atrial fibrillation and an increased long‐term risk of stroke. There is a relationship between the risk of stroke 6 months or more after surgical intervention and postoperative atrial fibrillation, according to two recent meta‐analyses involving 40 122 and 108 711 patients after coronary artery bypass grafting [4, 69]. Anticoagulation should lower the risk of these side effects if prolonged stroke and death are elevated as a result of postoperative atrial fibrillation recurrence. The fact that the included trials did not always report the anticoagulation rates before and after surgery may have an impact on the clinical outcomes of patients who develop atrial fibrillation after surgery. There is a paucity of information about anticoagulation's protective effect in patients with postoperative atrial fibrillation. The nonsurgical population, whose bleeding risk is dramatically different from postsurgical patients' and who may also have a very different trigger for atrial fibrillation, provides the majority of the evidence for anticoagulation in atrial fibrillation. There is only one study that looked at the connection between anticoagulation and mortality in patients who had heart surgery but had postoperative atrial fibrillation. After correcting for age, sex, and medical comorbidities, this observational study discovered a 22% relative death reduction in individuals receiving warfarin treatment [26]. Specific advice for starting anticoagulation after heart surgery is absent from major society guidelines on atrial fibrillation [3, 70]. To shed light on the therapy of this frequent complication, a randomized, placebo‐controlled trial of anticoagulation for postoperative atrial fibrillation in this cohort is needed. Pharmacological prophylaxis should be used more often to lower the risk of atrial fibrillation after cardiac surgery and its serious long‐term effects until more is known about the pathophysiology, risk factors, and effect of anticoagulation in atrial fibrillation after cardiac surgery.

## 4 Limitations

Given that a few of the researchers chosen for the meta‐analysis were not included, a variety bias might have occurred. Nevertheless, the excluded studies did not encounter the necessary standards to be incorporated into the meta‐analysis. Moreover, we did not have enough information to determine whether factors such as race and age had an impact on results. The effect of postoperative atrial fibrillation after cardiac surgery was the aim of the study. Bias may have increased as a result of the incorporation of incomplete or erroneous data from earlier studies. The individuals' age, gender, and race were likely sources of bias in addition to their nutritional status. Unintentionally skewed values might arise from incomplete data and unpublished research.

## 5 Conclusions

Individuals with cardiac surgery with postoperative atrial fibrillation had significantly higher mortality at 1 year, mortality at 5 years, mortality at 10 years, and overall stroke compared to those without postoperative atrial fibrillation. To validate this discovery, more studies are required, and thoughtfulness must be exercised when interrelating with its values. That would have an impact on how significant the evaluated assessments were.

## Ethics Statement

The authors have nothing to report.

## Consent

The authors have nothing to report.

## Conflicts of Interest

The authors declare no conflicts of interest.

## Supporting information

Supporting information.

Supporting information.

## Data Availability

On request, the corresponding author must provide access to the meta‐analysis database.
