# Supplementary material for: Effect of Postoperative Atrial Fibrillation After Cardiac Surgery: A Meta‐Analysis
Source: Clin Cardiol. 2024 Dec 4;47(12):e70053. doi: 10.1002/clc.70053 (PMC11617639; doi:10.1002/clc.70053)
Supplement: Supplementary file 2 — Supporting information. [file CLC-47-e70053-s001.docx]

**Supplemental Table 1.** Database Search Strategy for inclusion of examinations

| **Database** | **Search strategy** |
| --- | --- |
| **Google Scholar** | #1 "cardiac surgery" OR "mortality at 1 year"  #2 "mortality at 5 years" OR "mortality at 10 years" OR "postoperative atrial fibrillation" OR "overall stroke"  #3 #1 AND #2 |
| **Embase** | #1 'cardiac surgery' /exp OR 'mortality at 1 year' /exp OR 'postoperative atrial fibrillation'  #2 'mortality at 5 years'/exp OR 'mortality at 10 years'/exp OR 'overall stroke'  #3 #1 AND #2 |
| **Cochrane library** | #1 (cardiac surgery):ti,ab,kw (mortality at 1 year):ti,ab,kw (postoperative atrial fibrillation):ti,ab,kw (Word variations have been searched)  #2 (mortality at 5 years):ti,ab,kw OR (mortality at 10 years):ti,ab,kw OR(overall stroke):ti,ab,kw (Word variations have been searched)  #3 #1 AND #2 |
| **Pubmed** | #1 "cardiac surgery"[MeSH] OR "mortality at 1 year"[MeSH] OR "postoperative atrial fibrillation" [All Fields]  #2 "mortality at 5 years"[MeSH Terms] OR "mortality at 10 years"[MeSH] OR "overall stroke "[All Fields]  #3 #1 AND #2 |
| **OVID** | #1 "cardiac surgery"[All Fields] OR "mortality at 1 year" [All Fields] OR "postoperative atrial fibrillation" [All Fields]  #2 "mortality at 5 years"[ All fields] OR "mortality at 10 years"[All Fields] or "overall stroke"[All Fields]  #3 #1 AND #2 |

**Supplemental Table 2. Qualities of the chosen studies for the meta-analysis**

| **Study** | **Country** | **Total** | **With postoperative atrial fibrillation** | **Without postoperative atrial fibrillation** |
| --- | --- | --- | --- | --- |
| Villareal Rollo, 2004 [20] | USA | 6477 | 994 | 5483 |
| Ahlsson, 2009 [21] | Sweden | 1419 | 419 | 1000 |
| Filardo, 2009 [22] | USA | 6899 | 1814 | 5085 |
| Mariscalco, 2009 [23] | Sweden | 7621 | 1745 | 5876 |
| Ahlsson, 2010 [24] | Sweden | 271 | 165 | 106 |
| Bramer, 2010 [25] | Netherlands | 5098 | 1122 | 3976 |
| El-Chami, 2010 [26] | USA | 16169 | 2985 | 13184 |
| Filardo, 2010 [27] | USA | 1039 | 380 | 659 |
| Attaran, 2011 [28] | UK | 6556 | 3278 | 3278 |
| Bramer, 2011 [29] | Netherlands | 5098 | 1122 | 3976 |
| Girerd, 2012 [30] | Canada | 6728 | 1868 | 4860 |
| Helgadottir, 2012 [31] | Iceland | 744 | 326 | 418 |
| Saxena, 2012 [32] | Australia | 19497 | 5547 | 13950 |
| Barbieri, 2013 [33] | Brazil | 2628 | 326 | 2302 |
| Horwich, 2013 [34] | Canada | 8058 | 2214 | 5844 |
| O'Neal, 2013 [35] | USA | 13165 | 2907 | 10258 |
| Saxena, 2013 [36] | Australia | 2065 | 725 | 1340 |
| Weidinger, 2013 [37] | USA | 384 | 59 | 325 |
| Lee, 2014 [38] | Korea | 1171 | 244 | 927 |
| Thorén, 2014 [39] | Sweden | 7181 | 2512 | 4669 |
| Almassi, 2015 [40] | USA | 2096 | 549 | 1547 |
| Melduni, 2015 [41] | USA | 603 | 226 | 377 |
| Tsai, 2015 [42] | Taiwan | 266 | 126 | 140 |
| Konstantino, 2016 [43] | Canada | 4754 | 947 | 3807 |
| Schwann, 2018 [44] | USA | 9607 | 1992 | 7615 |
| Thorén, 2020 [45] | Sweden | 7145 | 2183 | 4962 |
| Taha, 2021 [46] | Sweden | 24523 | 7368 | 17155 |
| Lee, 2021 [47] | Korea | 507 | 94 | 413 |
| Almassi A, 2021 [48] | USA | 2103 | 551 | 1552 |
| Conen, 2021 [49] | Canada | 4624 | 778 | 3846 |
| Almassi B, 2021 [48] | USA | 2103 | 551 | 1552 |
| Kim, 2022 [50] | Korea | 1022 | 190 | 832 |
| Quin, 2022 [51] | USA | 2103 | 551 | 1552 |
| Oraii, 2022 [52] | Iran | 9310 | 8113 | 1197 |
| Alghosoon, 2023 [53] | Saudi Arabia | 1401 | 236 | 1165 |
| Rezk, 2023 [54] | Sweden | 7028 | 3121 | 3907 |
| Lilja, 2024 [55] | Sweden | 30870 | 10136 | 20734 |
| Herrmann, 2024 [56] | Sweden | 19443 | 1222 | 18221 |
|  | **Total** | **241299** | **68692** | **172607** |
